# Idiopathic Intracranial Hypertension: Pre- and Post-treatment Radiologic Analyses

**DOI:** 10.7759/cureus.75510

**Published:** 2024-12-10

**Authors:** José Diogo Martins, Pedro Mesquita, Joana Alves Costa, Iria Palma, João Carlos Costa

**Affiliations:** 1 Internal Medicine, Unidade Local de Saúde do Alto Minho, Viana do Castelo, PRT; 2 Medicine, University of Minho, Braga, PRT; 3 Internal Medicine, Unidade Local de Saúde de São José, Lisbon, PRT; 4 Radiology, JCC Diagnostic Imaging, Viana do Castelo, PRT; 5 Neurology, Clínica Girassol, Luanda, AGO

**Keywords:** cerebrospinal fluid dynamics, ct and mri brain, empty sella turcica, intracranial idiopathic hypertension, pseudotumor cerebri (ptc)

## Abstract

We discuss a case of a 19-year-old female who presented with pressure headaches and progressive vision loss. In the emergency department, a series of diagnostic tests were conducted, including CT, MRI, and lumbar puncture with measurement of opening pressure. All these examinations yielded results consistent with the suspected diagnosis of idiopathic intracranial hypertension (IIH). The patient was treated with acetazolamide to reduce intracranial pressure, resulting in the resolution of symptoms and the normalization of intracranial pressure on imaging studies. She was subsequently discharged from the hospital seven days after her initial presentation.

## Introduction

Idiopathic intracranial hypertension (IIH), historically known as “meningitis serosa” (1883) and “pseudotumor cerebri” (1904) [1], was defined as primary intracranial hypertension in 1955. Although initially considered benign due to minimal severe consequences, later cases of irreversible vision loss necessitated a change in terminology.

IIH manifests with non-specific symptoms due to increased intracranial pressure, including headache, nausea, pulsatile tinnitus, transient visual disturbances, and vision loss. Clinical findings may show diplopia (VI cranial nerve palsy) and papilledema. Comprehensive history and physical examination are crucial, alongside neuroimaging aligned with IIH diagnostic criteria [2]. Typical imaging findings involve an empty sella, lateral sinus collapse, posterior scleral flattening, optic nerve head protrusion, fully extended optic nerve sheaths, and optic nerve tortuosity. MRI is the preferred modality, though CT may reveal some signs. Lumbar puncture confirming opening pressure above 250 mmH₂O solidifies the diagnosis in obese patients [3].

While IIH is classified as “idiopathic” due to the absence of an identifiable cause, several theories attempt to explain the mechanism of increased intracranial pressure without evidence of a space-occupying lesion or hydrocephalus. Studies suggest a possible dysfunction in cerebrospinal fluid (CSF) reabsorption at the level of arachnoid granulations, which are responsible for draining CSF into the venous circulation. Reduced cerebral venous outflow, particularly in patients with stenosis of the lateral venous sinuses, is also considered a contributing factor, leading to secondary increases in CSF pressure and, consequently, intracranial hypertension. Inflammation or hypertrophy of the arachnoid granulations may partially obstruct the flow, exacerbating the elevated pressure.

## Case presentation

**Figure 1:**
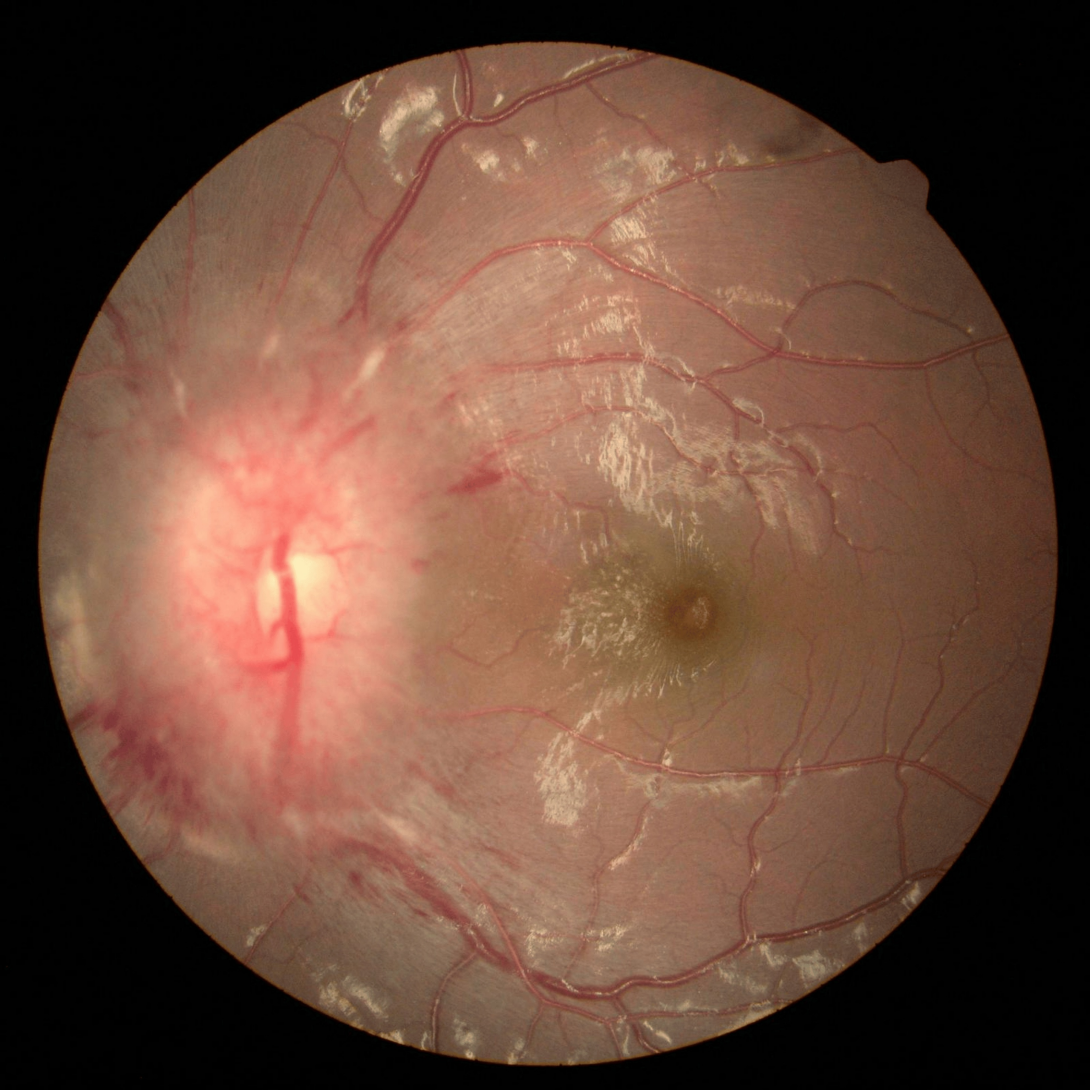
Fundus photograph of the left eye This fundus photograph illustrates grade 3 papilledema according to the Frisén scale. The optic disc shows significant elevation with obscuration of the major vessels as they cross the disc margin. Peripapillary nerve fiber layer swelling and hyperemia of the optic disc are evident, findings consistent with elevated intracranial pressure in IIH IIH: idiopathic intracranial hypertension

A 19-year-old female patient was referred to the emergency department due to complaints of pressure headaches and progressive vision loss. She described the headaches as severe, persisting for a week, and characterized by a progressive worsening with no relief from any specific factors. The patient denied any history of trauma, changes in daily routine, or variation with the time of day. Aside from reduced visual acuity, her physical and neurological examination was unremarkable. Ophthalmoscopy revealed grade 3 papilledema with no evidence of ischemic lesions or disc atrophy (Figure 1).

**Figure 2:**
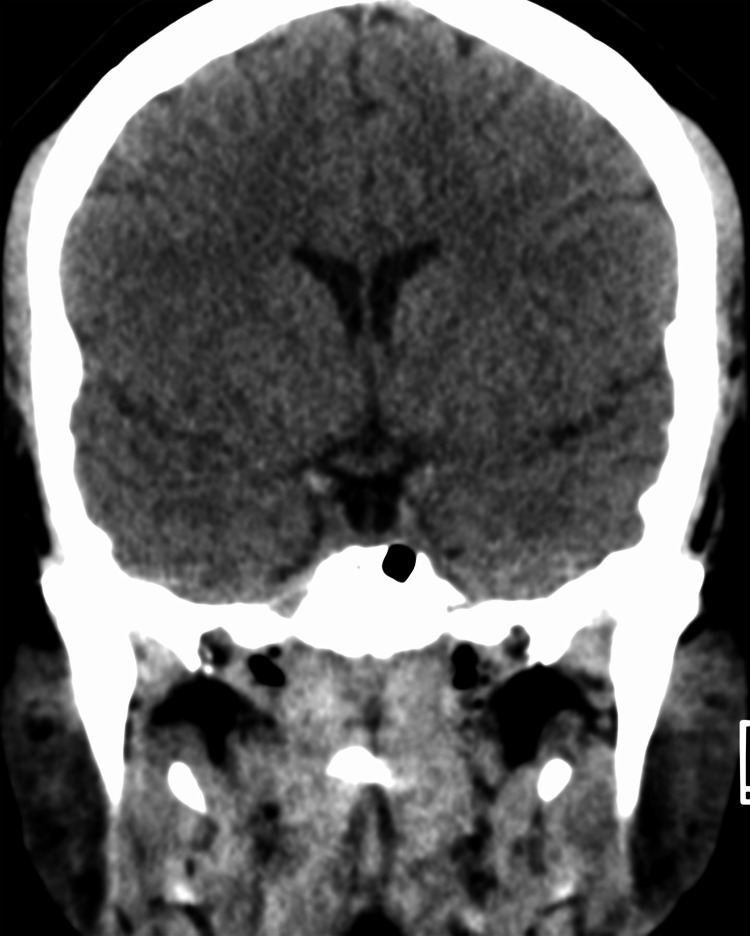
Coronal non-contrast CT image before treatment The image shows a partially empty sella sign with associated pituitary flattening and slight narrowing of the cavernous sinuses, consistent with IIH CT: computed tomography; IIH: idiopathic intracranial hypertension

**Figure 3:**
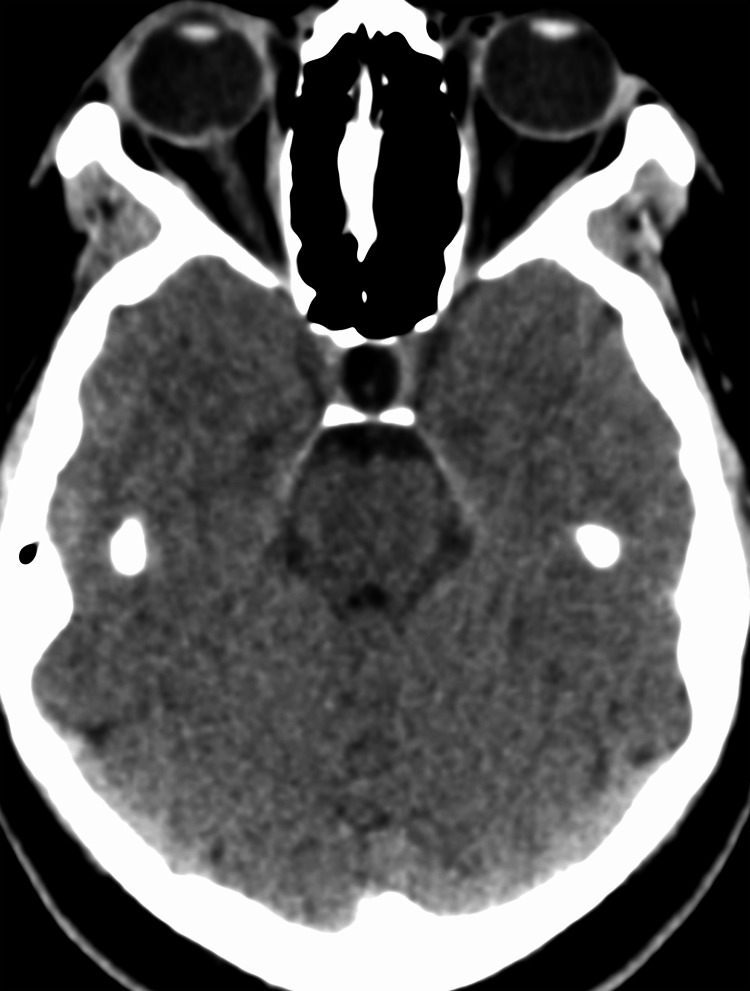
Axial non-contrast CT image before treatment The image demonstrates a partially empty sella, a radiological sign commonly associated with elevated intracranial pressure. Subtle protrusion of the optic nerve head can also be observed, indicating the effects of increased intracranial pressure CT: computed tomography

**Figure 4:**
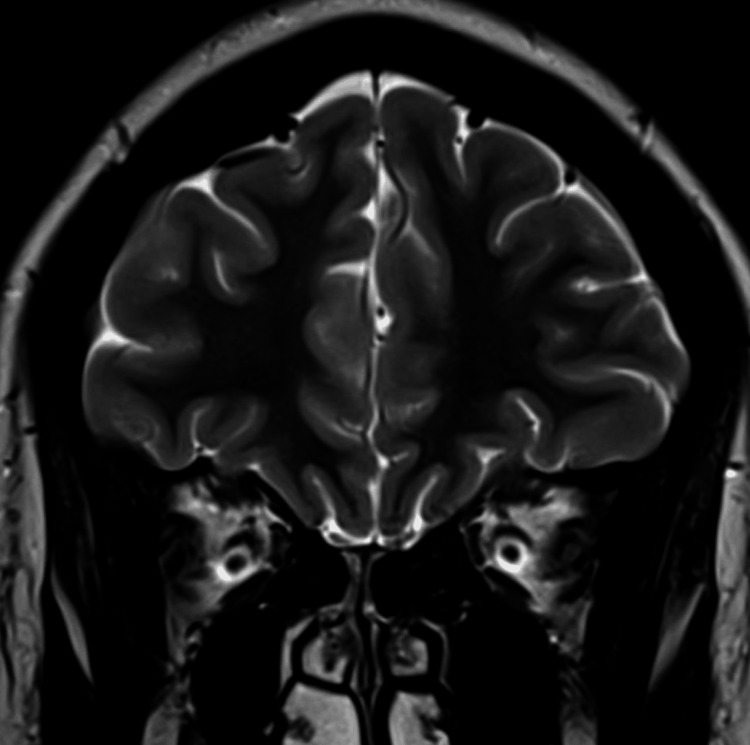
Distension of the perioptic subarachnoid in the coronal T2-weighted MRI (before treatment) The image shows the distension of the perioptic subarachnoid spaces MRI: magnetic resonance imaging

**Figure 5:**
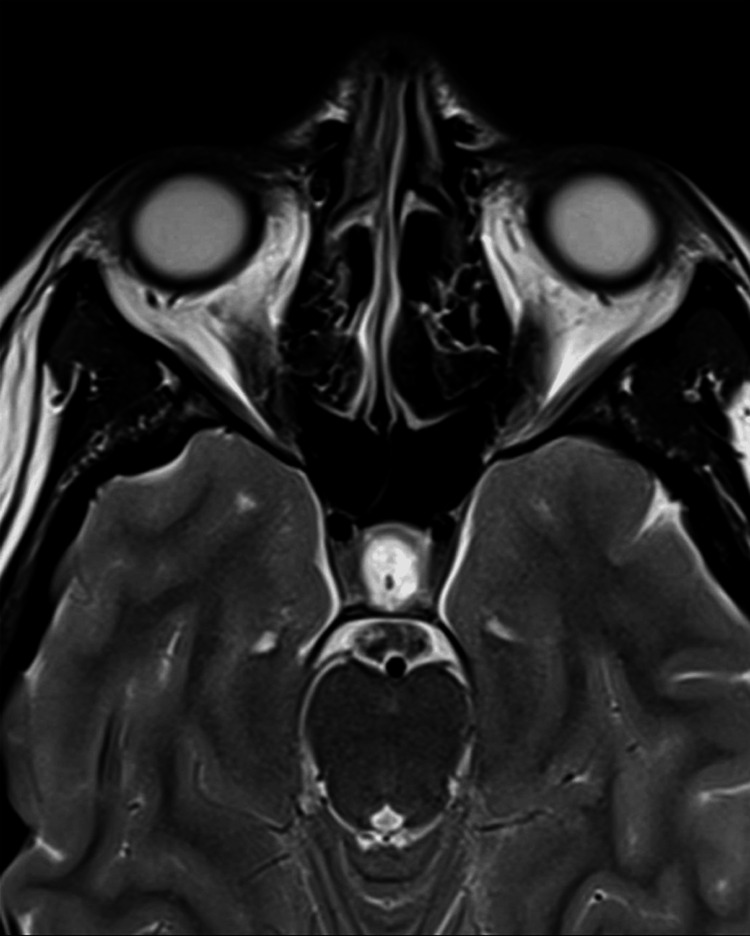
Empty sella in the axial T2-weighted MRI (before treatment) The image shows an empty sella sign with distension of the subarachnoid space within the sella turcica (Grade 3) MRI: magnetic resonance imaging

**Figure 6:**
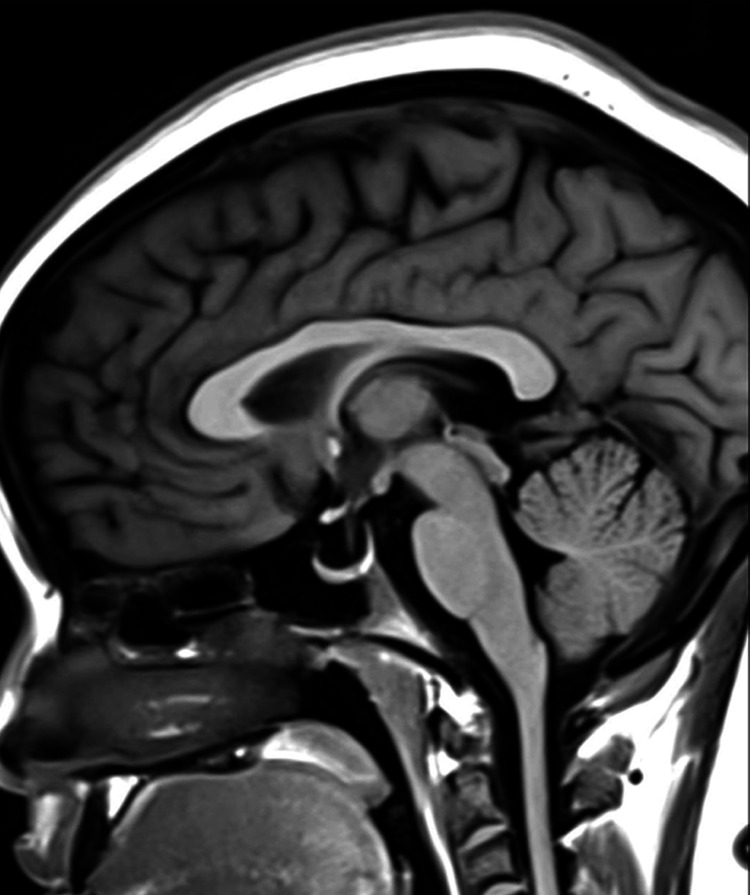
Empty sella in the sagittal T1-weighted MRI (before treatment) The image shows an empty sella sign (Grade 3–4) MRI: magnetic resonance imaging

**Figure 7:**
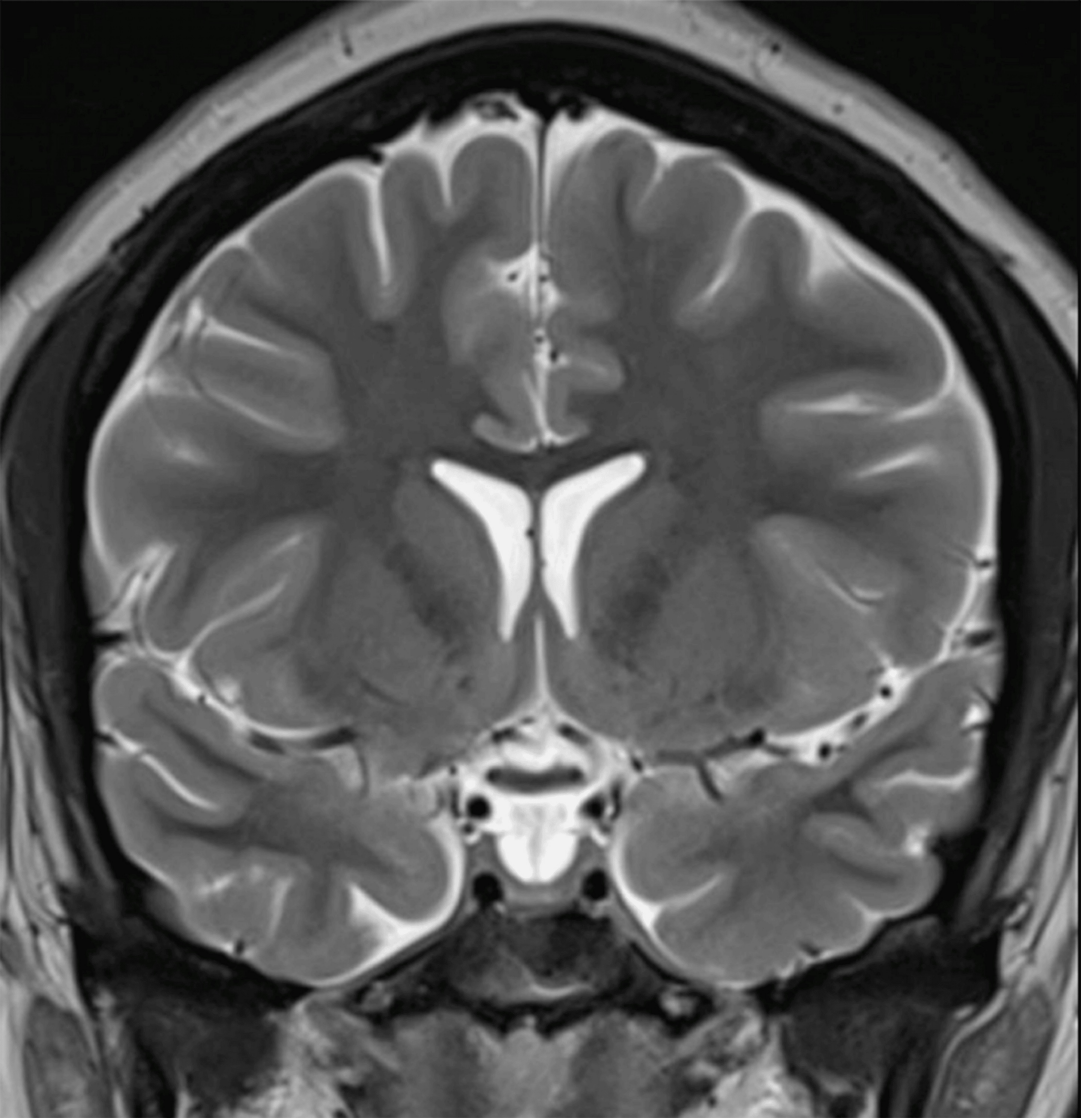
Pituitary flattening in the coronal T2-weighted MRI (before treatment) The image shows an empty sella sign with pituitary flattening against the sella turcica floor (Grade 3–4) and narrowing of the cavernous sinuses. No ventricular dilatation or intracranial mass lesion were identified MRI: magnetic resonance imaging

**Figure 8:**
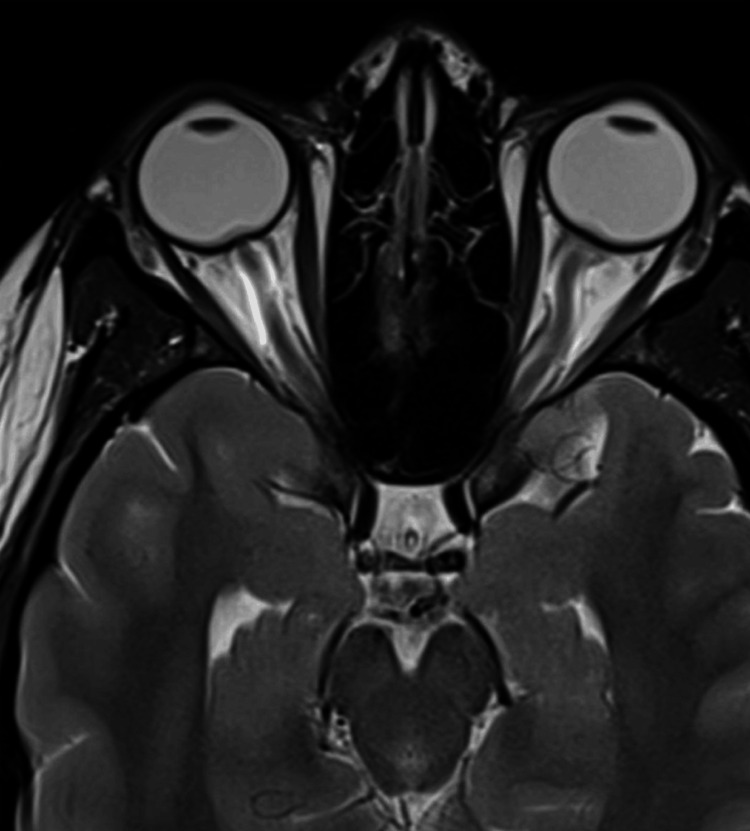
Papilledema and perioptic subarachnoid space ditension in the axial T2-weighted MRI (before treatment) The image shows papilledema, horizontal tortuosity, elongation of the optic nerve, distension of the perioptic subarachnoid space, and protrusion of the right optic nerve head MRI: magnetic resonance imaging

**Figure 9:**
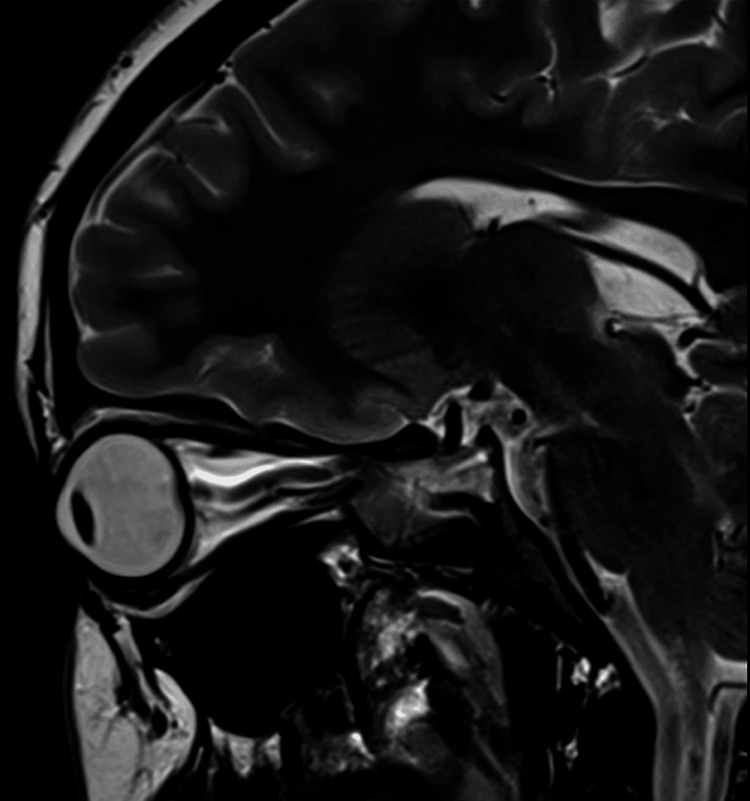
Papilledema and perioptic subarachnoid space ditension in the sagittal T2-weighted MRI (before treatment) The image of the left optic nerve shows flattening of the posterior sclera, vertical tortuosity and elongation of the optic nerve, distension of the perioptic subarachnoid space, and protrusion of the optic nerve head MRI: magnetic resonance imaging

A CT scan performed at admission (Figures 2-3) identified an empty sella and cavernous sinus collapse. To further investigate these findings, a 3 Tesla MRI (Figures 4-9) was performed, which ruled out secondary causes of intracranial hypertension and confirmed an empty sella sign (Grade 3) [4], posterior scleral flattening, horizontal tortuosity, elongation of the optic nerve, perioptic subarachnoid space distension, and protrusion of the optic nerve head. To definitively diagnose intracranial hypertension, a lumbar puncture was performed, revealing no CSF composition abnormalities (cell count: 3 leukocytes; protein: 32 mg/dL; glucose: 72 mg/dL), but an opening pressure of 321 mmH_2_O.

In light of these findings, the patient was admitted for clinical observation and treatment optimization until both clinical symptoms and imaging abnormalities resolved. The patient received intravenous acetazolamide at a dose of 500mg twice daily, resulting in a rapid improvement in symptoms. A thorough etiological investigation was conducted during hospitalization to identify potential secondary causes of intracranial hypertension. This included blood tests, which ruled out inflammatory, infectious, or endocrine disorders, and a CSF analysis, which excluded infections or malignancies. The comprehensive workup revealed no underlying secondary cause, supporting the diagnosis of IIH.

**Figure 10:**
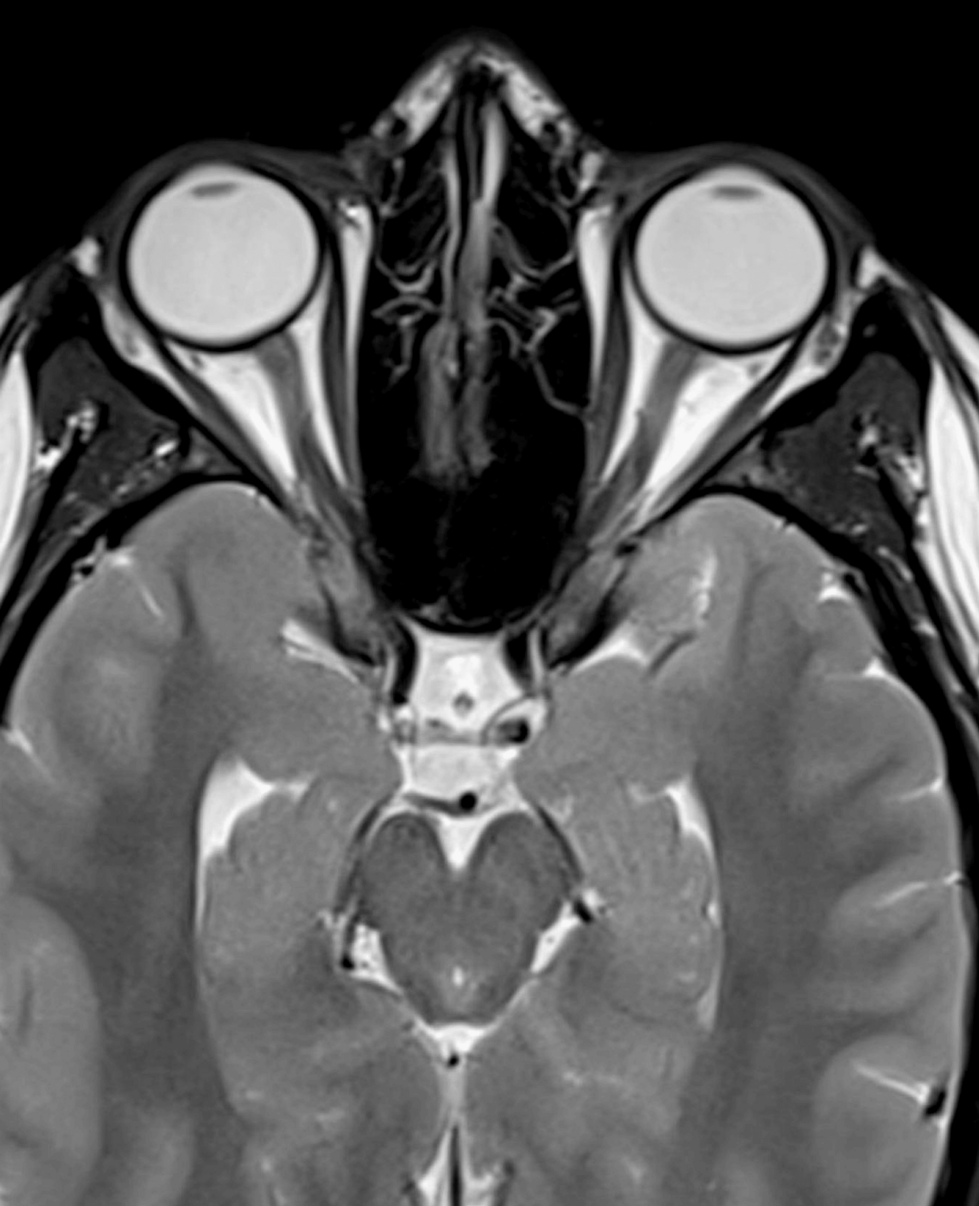
Normalization of perioptic subarachnoid space on the axial T1-weighted MRI (after treatment) The image shows complete resolution of bilateral perioptic subarachnoid space distension, with normal optic nerve and scleral morphology, the resolution of optic nerve head protrusion, and normalization of the perioptic subarachnoid spaces. The posterior sclera appears intact without flattening MRI: magnetic resonance imaging

**Figure 11:**
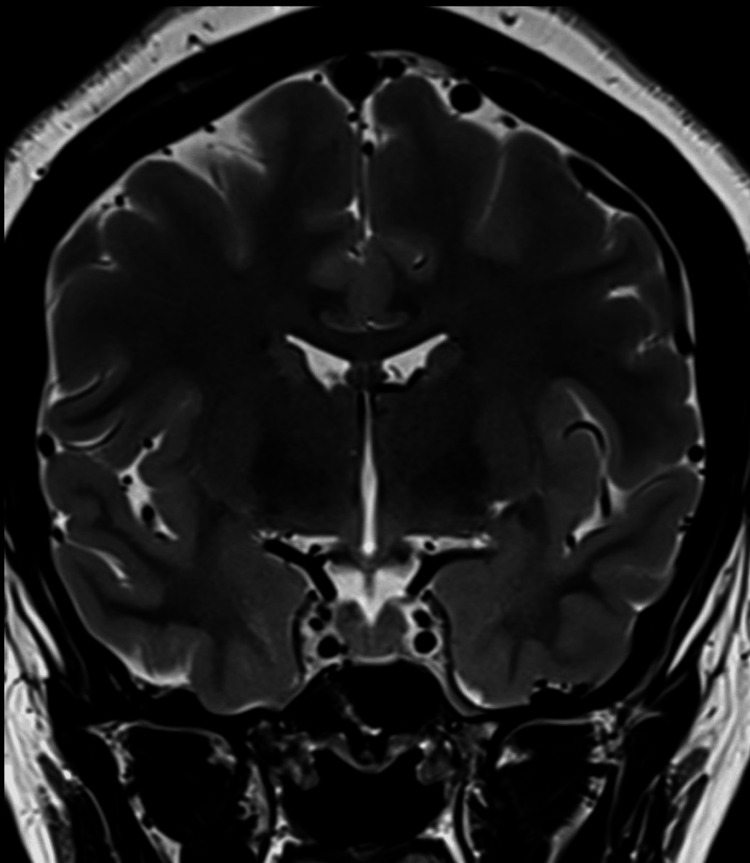
Normal sella turcica morphology in the coronal T2-weighted MRI (after treatment) The image shows normal sella turcica morphology, with the pituitary gland occupying the sella and no evidence of cavernous sinus narrowing MRI: magnetic resonance imaging

**Figure 12:**
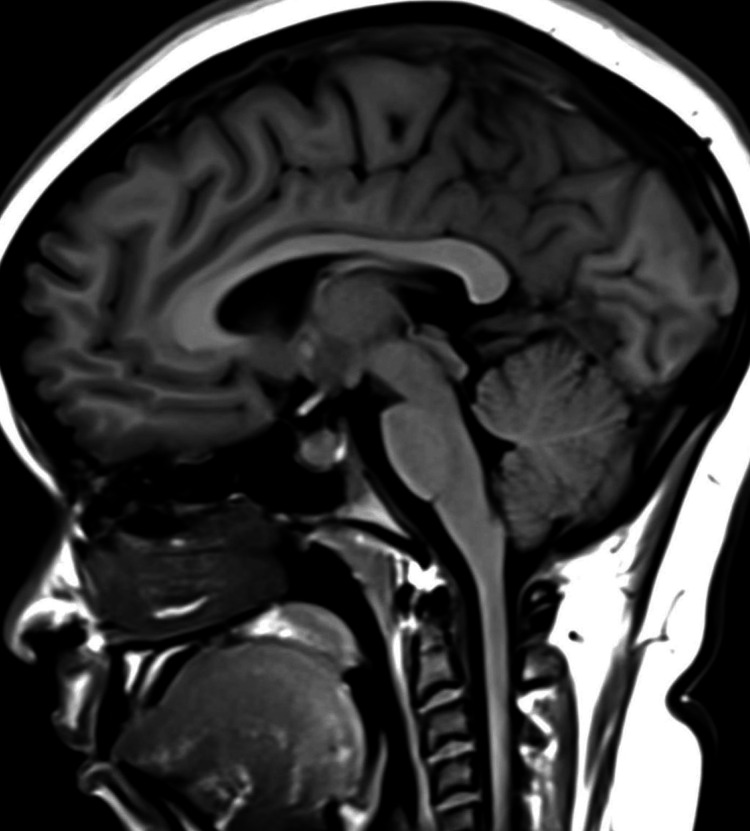
Normal sella turcica morphology in the sagittal T2-weighted MRI (after treatment) The image shows the resolution of the empty sella sign with normalization of the pituitary gland morphology MRI: magnetic resonance imaging

**Figure 13:**
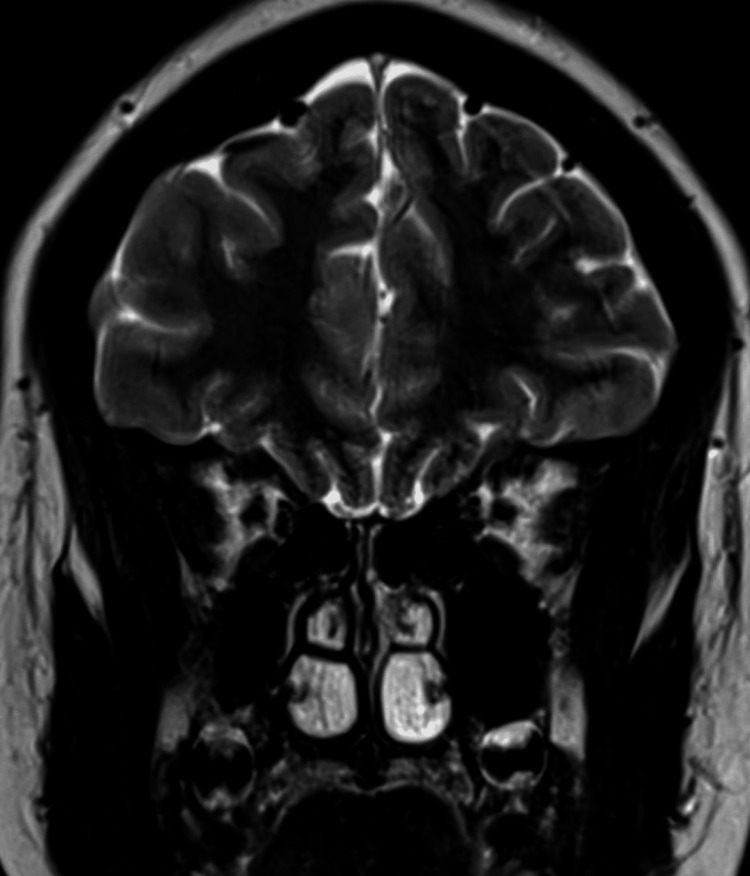
Restoration of perioptic subarachnoid spaces and optic nerve anatomy in the coronal T2-weighted MRI (after treatment) The image demonstrates normalization of the perioptic subarachnoid spaces and restoration of optic nerve anatomy (resolution of nerve head protrusion and normalization of the perioptic subarachnoid spaces) MRI: magnetic resonance imaging

A follow-up MRI on the seventh day of hospitalization demonstrated the normalization of all previously noted abnormalities (Figures 10-13).

## Discussion

The prevalence of IIH is notably higher in young women and those with obesity, indicating a strong association between the condition and hormonal and metabolic factors [5]. Endocrine disorders, such as hypoparathyroidism and hormone fluctuations related to steroid or growth hormone use, are also frequently associated with it [6]. Studies suggest that weight loss can improve symptoms and reduce intracranial pressure, making it an effective preventive measure in obese patients. Weight control programs and monitoring endocrine alterations in at-risk populations are recommended preventive interventions [7].

Acetazolamide remains the first-line treatment, as it reduces CSF production by inhibiting carbonic anhydrase [8]. The usual starting dose is 500-1000 mg per day, adjustable based on patient tolerance and clinical response. Side effects, such as paraesthesia and gastrointestinal symptoms, can limit long-term use. Other options include topiramate, which has a dual effect by promoting weight loss and decreasing CSF production. In refractory cases, surgical interventions, such as ventriculoperitoneal shunting or optic nerve sheath fenestration, are viable options to relieve pressure and protect vision [9].

The diagnosis of IIH relies on specific neuroimaging findings, with MRI being the preferred method. In recent years, MR venography (MRV) has become an essential tool, particularly for differentiating IIH from other causes of intracranial hypertension [10]. This technique enables detailed visualization of the venous sinuses, aiding in identifying stenoses that may contribute to venous outflow obstruction. The development of advanced MRI techniques with contrast has also improved diagnostic sensitivity for IIH and contributed to a more accurate assessment of treatment response.

## Conclusions

IIH predominantly affects young women, particularly those with obesity, underlining the importance of hormonal and metabolic factors in its pathogenesis. Weight loss and management of endocrine alterations are pivotal in both its prevention and treatment, while acetazolamide remains the cornerstone of medical therapy. Surgical options are available for refractory cases to preserve vision and reduce intracranial pressure. Accurate diagnosis hinges on advanced imaging modalities like MRI and MRV, which not only aid in ruling out secondary causes but also guide treatment decisions. The rising prevalence of IIH in regions with higher obesity rates highlights the need for public health initiatives focused on weight management and awareness campaigns about this condition.
